# The Enduring Table 1 Fallacy: A Meta-research Study of Baseline Testing in Anesthesiology and Pain Trials

**DOI:** 10.1097/ALN.0000000000005776

**Published:** 2025-09-25

**Authors:** Alessandro De Cassai, Burhan Dost, Esra Turunc, Engin İhsan Turan, Muzeyyen Beldagli, Mehmet Akif Yilmaz, Yunus Emre Karapinar, Annalisa Boscolo, Paolo Navalesi

**Affiliations:** 1 Department of Medicine, University of Padua, Padua, Italy; Institute of Anesthesia and Intensive Care Unit, University Hospital of Padua, Padua, Italy.; 2 Department of Anesthesiology and Reanimation, Ondokuz Mayis University Faculty of Medicine, Samsun, Turkey.; 3 Department of Anesthesiology and Reanimation, Ondokuz Mayis University Faculty of Medicine, Samsun, Turkey.; 4 Department of Anesthesiology and Reanimation, Kanuni Sultan Süleyman Education and Training Hospital, Istanbul Health Science University, Istanbul, Turkey.; 5 Department of Anesthesiology and Reanimation, Samsun Training and Research Hospital, Samsun, Turkey.; 6 Department of Anesthesiology and Reanimation, Ataturk University School of Medicine, Erzurum, Turkey.; 7 Department of Anaesthesiology and Reanimation, Istanbul University Cerrahpaşa Faculty of Medicine, Istanbul, Turkey.; 8 Department of Medicine, and Department of Cardiac, Thoracic, Vascular Sciences and Public Health, University of Padua, Padua, Italy; Institute of Anesthesia and Intensive Care Unit, University Hospital of Padua, Padua, Italy.; 9 Department of Medicine, University of Padua, Padua, Italy; Institute of Anesthesia and Intensive Care Unit, University Hospital of Padua, Padua, Italy.; 10 The article processing charge was funded by the University of Padua, Padua, Italy.

## Abstract

**Background:**

Randomized controlled trials (RCTs) are designed to achieve balanced distribution of baseline characteristics across study arms through random allocation, rendering null-hypothesis significance testing on these characteristics unnecessary and potentially misleading. Despite longstanding guidance discouraging this practice, its prevalence and patterns within anesthesiology and pain medicine literature remain unclear.

**Methods:**

The authors conducted a meta-research study of parallel-group RCTs published from 1996 to 2025 across 101 journals indexed under the “Anesthesiology and Pain Medicine” category in Scopus (Elsevier, The Netherlands). Data extraction included study characteristics, reporting of baseline testing, number of variables tested, and statistical significance. Multivariable logistic regression was used to identify factors associated with baseline testing, and a binomial test assessed whether the observed rate of significant findings exceeded the expected false-positive rate under the null hypothesis.

**Results:**

Of 2,453 eligible RCTs, 1,186 (48.3%) reported statistical testing of baseline characteristics. Among studies performing such testing, 228 (19.2%) reported at least one statistically significant difference, and 58 (25.4%) discussed it as a study limitation. A total of 11,516 variables were tested, with 424 (3.7%) reported as statistically significant—below the 5% expected by chance (*P* < 0.001). Larger author teams were associated with lower odds of baseline testing (odds ratio, 0.95; 95% CI, 0.93 to 0.97), while a higher number of variables tested increased the odds of finding at least one significant difference (odds ratio, 1.10; 95% CI, 1.07 to 1.12).

**Conclusions:**

Despite methodologic guidance and Consolidated Standards of Reporting Trials (CONSORT) recommendations, statistical testing of baseline characteristics remains common in anesthesiology RCTs and has not declined over time. This practice likely reflects persistent misunderstanding of randomization and may lead to misinterpretation of study validity. Education and stronger editorial policies are needed to align reporting behavior with best practices and improve trial transparency.

## Abstract 2

Despite methodologic guidance and CONSORT recommendations, statistical testing of baseline characteristics is common in the randomized controlled trials reported in the 101 anesthesiology and pain medicine journals in the Scopus database (Elsevier, The Netherlands), and this practice has not declined over time and was more common in journals with lower impact factors.

## Editor’s Perspective

**What We Already Know about This Topic**

- Randomized controlled trials are designed to achieve balanced distribution of baseline characteristics across study arms through random allocation, rendering null-hypothesis significance testing on these characteristics unnecessary and potentially misleading

**What This Article Tells Us That Is New**

- Despite methodologic guidance and Consolidated Standards of Reporting Trials (CONSORT) recommendations, statistical testing of baseline characteristics is common in the randomized controlled trials reported in the 101 anesthesiology and pain medicine journals in the Scopus database (Elsevier, The Netherlands), and this practice has not declined over time and was more common in journals with lower impact factors

Randomized controlled trials (RCTs) are designed to achieve balanced distribution of participant characteristics across study arms through the process of random allocation, minimizing the risk of systematic bias between groups. Nevertheless, it remains common practice for researchers to perform null-hypothesis significance testing on baseline characteristics in an attempt to determine whether groups are “balanced.” This approach is conceptually flawed.^1^ Although the purpose of conducting a statistical significance test is often to determine whether an observed difference is real or meaningful, the test actually evaluates the probability (the so-called “*P* value”) that the observed difference—or an even larger one—could have arisen purely by chance. However, in a properly randomized clinical trial, any differences between the two groups at baseline must, by definition, be due to chance because randomization eliminates systematic bias. Therefore, calculating the probability that a difference occurred by chance, when we already know it did, is logically inconsistent and ultimately meaningless.^2^ Even if this is a known aspect of RCTs and the use of statistical testing for baseline characteristics has been discouraged both in the Consolidated Standards of Reporting Trials (CONSORT) reporting guidelines as “it is superfluous and can mislead investigators and their readers”^3^ and in several articles and statistical commentaries within the anesthesiology literature advocating against its use,^4–6^ it is not uncommon.

This misuse reflects a misunderstanding of the purpose of randomization and may lead to misinterpretation of trial validity. Sherry *et al.*^7^ performed a large analysis of phase III oncology trials and found that 25% of trials included significance testing of baseline demographic and clinical characteristics, calling it “table 1 fallacy.”

In response to persistent misuse of baseline testing, our study’s primary aim was to quantify the prevalence of baseline characteristics that are reported and statistically tested in parallel RCTs within anesthesia and pain medicine literature.

Our secondary aims were to assess the frequency and patterns of baseline statistical testing between randomized groups, quantify the prevalence of statistically significant baseline differences, and investigate factors associated with the likelihood of reporting such differences, including the number of baseline variables tested, sample size, and number of groups. Additionally, the study aimed to determine whether the observed proportion of statistically significant baseline differences exceeded the expected 5% false-positive rate under the null hypothesis of no real differences.

## Materials and Methods

To identify relevant sources, we included all journals assigned a CiteScore in 2024 within the Anesthesiology and Pain Medicine category of the Scopus database (Elsevier, The Netherlands), ensuring the inclusion of peer-reviewed journals recognized within this specialty. The Scopus database was subsequently queried to retrieve all indexed RCTs. See Supplemental Digital Content 1 (https://links.lww.com/ALN/E255) for the full search strategy. Retrieved records were manually and independently screened by four members of the research team to identify studies meeting the predefined inclusion criteria. We included all RCTs from 1996 to date with parallel group design while excluding cluster-randomized trials, crossover studies, and studies not published in English.

From the included studies, we extracted the following bibliographic variables: number of authors, publication year, journal name, journal quartile at the time of publication, and number of citations. Journal quartile is a percentile-based ranking of a journal within its subject category (in this case, anesthesiology) of the annual *Journal Citation Reports*,^8^ determined by its impact factor. Journals are divided into four equal groups: the top 25% (quartile [Q] 1), 26 to 50% (Q2), 51 to 75% (Q3), and 76 to 100% (Q4). For the purposes of this analysis, we arbitrarily grouped Q3, Q4, and journals without an assigned impact factor into a single category. Additionally, since the 2025 impact factor and corresponding quartile are not yet available, we used the 2024 journal quartile as a proxy for trials published in 2025. Additionally, the following data were retrieved from the full texts: number of groups, randomization methods, total number of patients, whether baseline characteristics were statistically tested (yes/no), total number of baseline variables reported, number of baseline variables found to be statistically significant (if tested), and whether any statistically significant baseline differences were discussed as potential study limitations. Although statistical testing of baseline characteristics is usually presented in the first table of a RCT, we examined the entire Results section to retrieve this information and did not limit our assessment to table 1 alone.

### Statistical Analysis

Continuous variables were summarized as median (first quartile, third quartile) [minimum to maximum], while categorical variables were reported as numbers (percentages). Differences between the two groups were tested using the Mann–Whitney U test for continuous variables and the chi-square test or Fisher exact test for categorical variables, as appropriate.

To estimate the 95% CI for the median proportions (journals that have always, never, or partially analyzed baseline characteristics), we used nonparametric bootstrapping with 10,000 resamples. A multivariable logistic regression model was used to explore the association between selected independent variables and the binary outcome variable. A simple linear regression model was used to assess the temporal trend in the number of published RCTs over time. Results were expressed as odds ratios (ORs) with 95% CIs and corresponding *P* values. Before model fitting, all independent variables were tested for multicollinearity using the variance inflation factor. A variance inflation factor greater than 5 was considered indicative of potential multicollinearity.

To assess whether the observed proportion of statistically significant variables significantly differed from the expected proportion of 0.05 (5%), a two-sided binomial test was conducted. This test evaluates the null hypothesis that the true probability of success is equal to a specified value—in this case, 0.05—against the alternative hypothesis that it is different. Additionally, a 95% CI for the observed proportion was calculated to estimate the range within which the rate likely falls.

For all statistical tests, a two-sided *P* value < 0.05 was considered statistically significant.

All analyses were conducted using R (R Foundation for Statistical Computing, Vienna, Austria., https://www.R-project.org/) version 4.4.2 (“Pile of Leaves”) and using the packages Rcmdr,^9^ ggplot2,^10^ and dplyr.^11^

## Results

### Study Selection

**Fig. 1.**
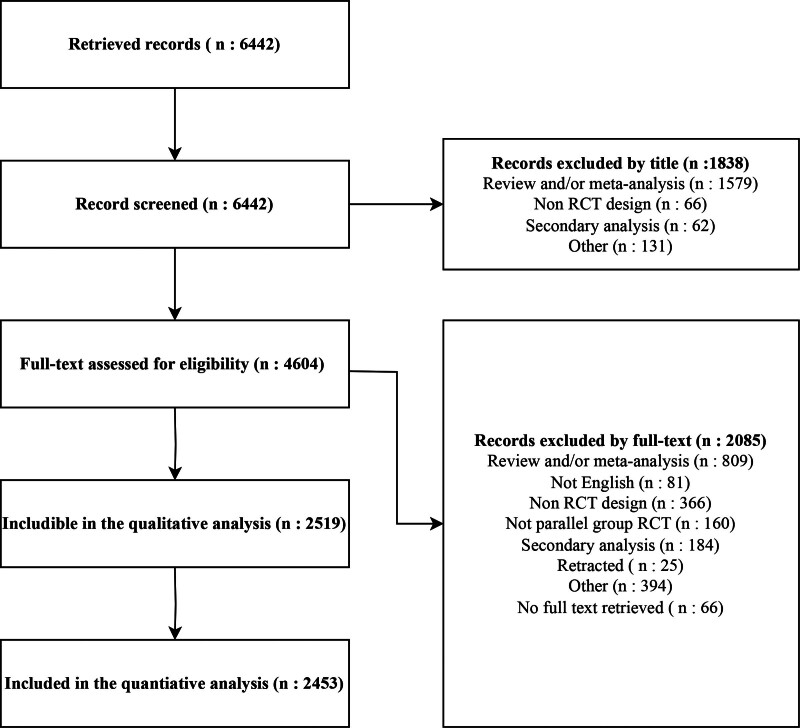
Flow chart for study inclusion and exclusion. Other refers to editorial, letter, comment, position paper, or guidelines. RCT, randomized controlled trial.

The search was conducted on May 5, 2025, and the study inclusion and exclusion process is illustrated in figure 1. Briefly, 6,442 records in total were identified through the initial search. Of these, 1,838 were excluded based on title screening, and 4,604 full-text articles were assessed for eligibility. Full texts could not be retrieved for 66 records. Among the remaining studies, 2,019 were excluded—primarily because they were review articles (with or without meta-analyses), employed non-RCT study designs, or represented secondary analyses of previously published trials. Additionally, 160 RCTs were excluded due to their crossover or cluster design, leaving a total of 2,519 RCTs for inclusion.

However, in 66 trials, baseline data were not reported or were only generically stated as “comparable,” limiting our possibility to assess whether statistical tests on baseline characteristics were performed. As a result, 2,453 RCTs were finally included for quantitative analysis.

### Study Characteristics

**Fig. 2.**
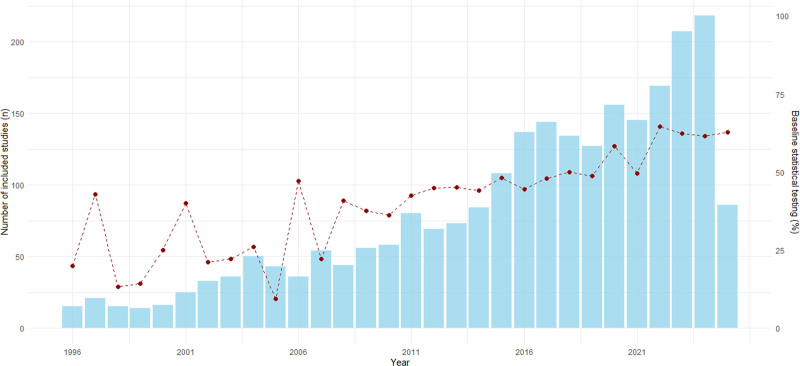
Annual number of included studies and proportion of baseline statistical testing. *Bars* represent the number of included studies per year (*left* y*-axis*). The *red dashed line* with markers represents the proportion of studies reporting baseline statistical testing (*right* y*-axis*).

We observe a significant increase in the number of included RCTs over time as shown by a linear regression model with an estimated average growth of 6.08 RCTs per year (95% CI, 5.03 to 7.14; *P* < 0.001; fig. 2).

The 2,453 RCTs included in the quantitative analysis were published across 101 different journals, with a median of 8 articles per journal (interquartile range, 3 to 32). A detailed list of the number of included RCTs per journal is available in Supplemental Digital Content 1 (https://links.lww.com/ALN/E255). A total of 744 articles were published in journals in the first quartile, 547 in journals in the second quartile, and 1,162 in journals in the third quartile, in the fourth quartile, or in journals without an impact factor.

### Prevalence and Study-level Correlates of Baseline Statistical Testing

Of the included trials, statistical testing on baseline characteristics between the groups was performed in 1,186 studies (48.3%). Articles from journals with consistent policies accounted for only a minority of the total (72 articles; 2.9%). The overall median usage was 58% (95% CI, 50 to 67%), which decreased to 50% (95% CI, 45.5 to 58%) when considering only journals with mixed reporting.

The characteristics of studies that performed statistical testing on baseline variables differed significantly from those that did not, as shown in table 1. Although the medians of several variables were similar between groups, statistical testing identified significant differences—likely reflecting variation in the tails of the distributions, as indicated by differences in the first and third quartiles, as well as the minimum and maximum values.

**Table 1.** Comparison of Studies Testing *versus* Not Testing Baseline Characteristics: Univariate Analysis

Variable	Baseline Characteristics Statistically Tested		*P* Value
	No	Yes	
Year	2016 (2009–2021) [1996–2025]	2019 (2015–2023) [1996–2025]	< 0.001
Journal quartile			
First	490 (65.8%)	254 (34.2%)	< 0.001
Second	302 (55.2%)	245 (44.8%)	
Other	475 (40.9%)	687 (59.1%)	
Groups	2 (2–2) [2–8]	2 (2–2) [2–6]	< 0.001
Authors	6 (5–8) [1–211]	6 (4–8) [1–35]	< 0.001
Study population	87 (50–148) [8–3,984]	75 (51–115) [10–18,836]	< 0.001
No. of variables	8 (5.25–13) [1–125]	8 (6–12) [1–58]	0.039
No. of citations	21 (7.25–44) [0–686]	11 (2–29) [0–393]	< 0.001

Continuous data are expressed as median (first–third quartile), [minimum–maximum]. Categorical data are expressed as numbers (percentage). “Year” indicates the year of publication; “groups” refers to the number of groups analyzed in the trial; “authors” denotes the number of authors of the article; “study population” represents the sample size included in the trial; “number of variables” corresponds to the number of baseline characteristics reported in the article; “number of citations” refers to the number of times the trial was cited according to the Scopus database.

RCTs that reported statistical testing of baseline characteristics were generally more recent and published in journals in lower quartiles, involved fewer authors, compared fewer groups within the same trial, and assessed fewer baseline variables. While the total sample size also appeared significantly lower in the bivariate comparison (table 1), this association was not confirmed in the multivariable logistic regression analysis (table 2). A sensitivity analysis was performed, restricting the dataset by omitting journals in which baseline testing practices did not vary. The rationale was that in journals with consistent policies, the journal itself could be the primary determinant. This analysis (Supplemental Digital Content 1, https://links.lww.com/ALN/E255) provided results similar to those from the main analysis.

**Table 2.** Logistic Regression Analysis Identifying Risk Factors for Baseline Characteristics Assessment

Variable	OR (95% CI)	*P* Value	Multicollinearity (VIF)
Year	1.07 (1.06–1.09)	< 0.001	1.04
Journal quartile			1.02
First quartile	Reference		
Second quartile	1.43 (1.13–1.81)	0.003	
Other quartiles	2.31 (1.88–2.84)	< 0.001	
Groups	0.74 (0.65–0.85)	< 0.001	1.02
Authors	0.95 (0.93–0.97)	< 0.001	1.08
Study population	1.00 (1.00–1.00)	0.301	1.01
No. of variables	0.98 (0.97–1.00)	0.017	1.09

Baseline characteristics statistical testing as the dependent variable. “Year” indicates the year of publication; “groups” refers to the number of groups analyzed in the trial; “authors” denotes the number of authors of the article; “study population” represents the sample size included in the trial; and “number of variables” corresponds to the number of baseline characteristics reported in the article.

OR, odds ratio; VIF, variance inflation factor.

Interestingly, study not testing for baseline characteristics accounted for more citations (table 1).

### Occurrence and Interpretation of Statistically Significant Baseline Differences

Among the trials that performed statistical testing of the baseline characteristics, 228 trials (19.2%) reported at least one statistically significant difference at baseline, and of those, 58 (25.4%) explicitly discussed this difference as a possible limitation of the study.

Across all trials conducting baseline testing, a total of 11,516 variables were analyzed, of which 424 (3.7%) were reported as statistically significant. In a logistic regression model, studies that tested a higher number of variables were more likely to report at least one statistically significant result (OR, 1.10; 95% CI, 1.07 to 1.12; *P* < 0.001). In contrast, no significant association was found for the number of patients included in the study (OR, 1.00; 95% CI, 1.00 to 1.00; *P* = 0.125) or for the number of groups compared (OR, 1.24; 95% CI, 0.94 to 1.60; *P* = 0.114).

Results from the binomial test indicated that the observed proportion of significant variables (3.7%) was statistically lower than the expected 5% under the null hypothesis (95% CI, 3.3 to 4.0%; *P* < 0.001), suggesting that the number of significant results was less than what would be expected by chance alone.

## Discussion

The main finding of this large-scale analysis of RCTs published in anesthesia and pain medicine journals is that nearly half (48.3%) reported statistical significance testing of baseline characteristics. We expected to observe a decline in this practice over time; however, the percentage of studies performing baseline significance testing not only aligns with rates reported nearly 30 yr ago but appears to be increasing.^12^ Although it is reassuring that journals in the highest quartiles are less affected by this phenomenon—suggesting that these journals likely have editors, peer reviewers, and overall policies that are more aware of the issue and ensure greater oversight of published articles—this practice persists despite recommendations against it, including those from the CONSORT guidelines,^3^ likely reflecting a lack of awareness among authors about this issue. This also underscores the importance of the present report.

Editors and peer reviewers act as gatekeepers to prevent inappropriate statistical testing of baseline characteristics in RCTs. However, it must be acknowledged that authors, reviewers, and editors are not distinct populations but rather the same scientific community acting in different roles. Consequently, if awareness of the issue is limited among authors, it is reasonable to assume that a proportion of reviewers and editors may also lack awareness. This may explain why inappropriate testing persists despite guidance. In line with this interpretation, we observed that journals in the highest quartiles—likely benefiting from more experienced editorial boards and reviewer pools—had the lowest rates of statistical testing.

On the one hand, authors should be aware that properly performed randomization balances both known and unknown confounding factors^13^ given that the included population is adequately large; some studies have suggested that a minimum of 1,000 patients may be necessary to achieve true balance between trial arms^14,15^; on the other hand, even small differences that are not statistically significant may still be clinically meaningful. For example, a difference in the proportion of diabetic patients—30% in one group *versus* 15% in another—may not reach statistical significance but should still be considered in a study evaluating stroke risk, as diabetes is a major risk factor for this condition.^16^ For this reason, reporting of absolute difference or standardized difference could be more helpful, especially if accompanied by a critical discussion of the authors^17^ and in case of identified, potentially relevant differences, the use of subgroups and sensitivity analysis could be useful to investigate the effect of such difference.^1^

However, it has been proposed that baseline assessment could serve as a red flag for possible issues such as incorrect randomization^18^ or scientific fraud,^19^ as it may indicate imbalances too extreme to be due to chance alone. However, because such analyses are easily misinterpreted and may mislead readers, this evaluation should remain part of the authors’ internal quality checks and editorial review and it should not be reported in the manuscript.

Interestingly, studies involving a larger number of authors were less likely to report such testing. While we interpret this as potentially reflecting a higher likelihood that at least one member of a larger research team identified and discouraged the practice, another possible explanation is that larger teams may involve more experienced researchers or statistical experts who are more familiar with best reporting practices and current guidelines.

The proportion of baseline variables found to be statistically significant (3.7%) was lower than the 5% expected under the null hypothesis, suggesting either underreporting or selective reporting of significant findings, a bias not previously identified in scientific publishing to our knowledge but that raises several concerns as investigators may have conducted multiple statistical tests but presented only nonsignificant baseline results in order to avoid drawing attention to perceived shortcomings in randomization. Furthermore, the likelihood of identifying at least one significant difference increased with the number of variables tested (OR, 1.10), but not with sample size or number of groups—findings that align with statistical theory and support the “multiple testing” concern often raised in baseline comparisons.

Our study has several limitations and some points of strength that we would like to briefly discuss.

A major strength of this study is its comprehensive scope: we analyzed a large and representative sample of RCTs across 101 anesthesiology and pain medicine journals using rigorous, prespecified inclusion criteria and standardized data extraction by multiple independent reviewers, enhancing the generalizability of our findings across the specialty. We also used robust statistical approaches, including regression modeling and bootstrapped CI, to quantify associations and uncertainty.

However, several limitations must be acknowledged. First, despite our search strategy, we may have missed relevant studies, particularly those published in non-English languages or in journals not indexed in Scopus. Second, we were unable to assess author intent, peer reviewer reports, or editorial policy, which may have influenced reporting behavior. Third, while we could count the number of baseline variables and detect statistical testing, we could not evaluate the clinical importance of reported differences—a limitation inherent to quantitative meta-research. Fourth, this study was not preregistered, which may increase the risk of bias in study selection, analysis, and reporting. Fifth, we must acknowledge that we may have underestimated the prevalence of baseline statistical testing, and the actual rate of researchers employing this practice could be higher than reported in this study due to publication bias. Trials exhibiting substantial baseline imbalances may have been less likely to be published, particularly if such imbalances were perceived negatively during the peer review process. Finally, the observational design precludes causal inference. Associations between study features and baseline testing practices should be interpreted as descriptive rather than explanatory.

### Conclusions

Despite clear guidance to the contrary, statistical testing of baseline characteristics remains common in anesthesia and pain medicine RCTs. This practice is unevenly distributed across journals and inconsistently interpreted by authors. Our findings highlight a persistent gap between methodologic best practices and actual reporting behavior, underscoring the need for education and policy reinforcement to improve the transparency and integrity of RCT reporting in our field.

### Research Support

Support was provided solely from institutional and/or departmental sources.

### Competing Interests

Dr. Navalesi has received research grants and/or equipment support from Gilead (Foster City, California), Mindray (Shenzhen, Guangdong, China), Fisher & Paykel (Auckland, New Zealand), and Intersurgical SpA (Mirandola, Italy). He receives royalties from Intersurgical SpA for the invention of the Helmet Next, and has received lecturing fees from Getinge (Gothenburg, Sweden), Mindray, Philips (Amsterdam, The Netherlands), Gilead, GSK (London, United Kingdom), Shionogi (Osaka, Japan), and Intersurgical SpA. The other authors declare no competing interests.

## Supplemental Digital Content

Supplemental Digital Content 1, https://links.lww.com/ALN/E255

## Supplementary Material


