# Supplementary material for: The Enduring Table 1 Fallacy: A Meta-research Study of Baseline Testing in Anesthesiology and Pain Trials
Source: Anesthesiology. 2025 Sep 25;144(1):156–62. doi: 10.1097/ALN.0000000000005776 (PMC12677328; doi:10.1097/ALN.0000000000005776)
Supplement: Supplementary file 1 [file aln-144-156-s001.pdf]

The Enduring Table 1 Fallacy: A Meta-Research Study of Baseline Testing in Anesthesiology and Pain Trials

SDC

**Search Strategy**	**p. 2**
**Included randomized controlled trials per journal**	**p. 3**
**Sensitivity analysis**	**p. 9**

Search strategy

SRCID (21410) OR SRCID (21858) OR SRCID (18148) OR SRCID (16714) OR
SRCID (21846) OR SRCID (21843) OR SRCID (11000153716) OR SRCID (19783)
OR SRCID (16790) OR SRCID (21869) OR SRCID (21948) OR SRCID (4100151514)
OR SRCID (16484) OR SRCID (13222) OR SRCID (21100889709) OR SRCID (21910
) OR SRCID (21887) OR SRCID (19700182810) OR SRCID (21100435142) OR SRCID
(21100854841) OR SRCID (18500161700) OR SRCID (17973) OR SRCID (15987) OR
SRCID (19700175077) OR SRCID (19700174806) OR SRCID (21911) OR SRCID (
14995) OR SRCID (21938) OR SRCID (4400151515) OR SRCID (145429) OR SRCID
(14880) OR SRCID (21100199779) OR SRCID (21909) OR SRCID (18153) OR
SRCID (21100394091) OR SRCID (21100307455) OR SRCID (21871) OR SRCID (
23831) OR SRCID (27523) OR SRCID (21100313904) OR SRCID (19700174903) OR
SRCID (21921) OR SRCID (21100878009) OR SRCID (15872) OR SRCID (21397)
OR SRCID (19700187304) OR SRCID (15989) OR SRCID (21101050348) OR SRCID (
52092) OR SRCID (21100849723) OR SRCID (21855) OR SRCID (19500157041) OR
SRCID (21100773822) OR SRCID (21934) OR SRCID (19700172216) OR SRCID (
4700152761) OR SRCID (21100255120) OR SRCID (71588) OR SRCID (21100944389
) OR SRCID (21411) OR SRCID (21100370880) OR SRCID (21100944647) OR SRCID
(21101166816) OR SRCID (21101126637) OR SRCID (21949) OR SRCID (
21101152881) OR SRCID (21101173097) OR SRCID (18844) OR SRCID (21907) OR
SRCID (19700200839) OR SRCID (19700201145) OR SRCID (21950) OR SRCID (
21100976171) OR SRCID (21101073950) OR SRCID (7200153146) OR SRCID (
4000151502) OR SRCID (23060) OR SRCID (12400154726) OR SRCID (21101052864
) OR SRCID (21100981226) OR SRCID (21101201218) OR SRCID (34839) OR SRCID
(21101097212) OR SRCID (21894) OR SRCID (21100445638) OR SRCID (
21101041559) OR SRCID (26729) OR SRCID (21101107941) OR SRCID (
21101041554) OR SRCID (21401) OR SRCID (21101038514) OR SRCID (
21100909460) OR SRCID (17616) OR SRCID (21100831489) OR SRCID (5300152215
) OR SRCID (24289) OR SRCID (21420) OR SRCID (19700174980) OR SRCID (
21101042200) OR SRCID (21876) OR SRCID (21101050071) OR SRCID (21394) OR
SRCID (19700175249) OR SRCID (21100875939) OR SRCID (21101192740) OR
SRCID (5100152404) OR SRCID (21962) OR SRCID (33502) OR SRCID (
21101082212) OR SRCID (19905) OR SRCID (21101042491) OR SRCID (
21101115808) OR SRCID (19700174976) OR SRCID (19700174634) OR SRCID (
21951) OR SRCID (21101091059) OR SRCID (21101169013) OR SRCID (
21101060899) OR SRCID (4000148111) OR SRCID (14300154708) OR SRCID (12576
) OR SRCID (20434) OR SRCID (12578) OR SRCID (21101186835) OR SRCID (
21974) OR SRCID (66252) OR SRCID (21435) OR SRCID (21920) OR SRCID (
21101151823) OR SRCID (21395) OR SRCID (21101153196) OR SRCID (
21101034336) OR SRCID (4000150801) OR SRCID (12545) OR SRCID (21101152065
) OR SRCID (21101199900) AND TITLE-ABS-KEY ("randomized controlled trial") OR
TITLE-ABS-KEY ("randomized clinical trial") OR TITLE-ABS-KEY ("randomized trial")
AND PUBYEAR > 1995 AND PUBYEAR < 2026

Included randomized controlled trials per journal

Journal	*Included RCT(n)*
Anesthesia and Analgesia	166
BMC Anesthesiology	156
British Journal of Anaesthesia	138
Anesthesiology	125
Pain	115
Journal of Clinical Anesthesia	106
Journal of Cardiothoracic and Vascular Anesthesia	104
European Journal of Anaesthesiology	92
Acta Anaesthesiologica Scandinavica	82
Clinical Journal of Pain	75
Journal of Pain and Symptom Management	67
Anaesthesia	58
Egyptian Journal of Anaesthesia	58

Journal of Pain Research	53
Pain Medicine (United States)	52
Journal of Anesthesia	49
Anesthesiology and Pain Medicine	48
European Journal of Pain (United Kingdom)	48
Canadian Journal of Anesthesia	44
Journal of Pain	43
Pain Physician	43
Indian Journal of Anaesthesia	41
Regional Anesthesia and Pain Medicine	40
Minerva Anestesiologica	39
Anaesthesia, Pain and Intensive Care	37
Korean Journal of Anesthesiology	32
Journal of Anaesthesiology Clinical Pharmacology	30
Paediatric Anaesthesia	27

Journal of Clinical Monitoring and Computing	25
Pain Practice	24
Anaesthesia and Intensive Care	23
Anaesthesia Critical Care and Pain Medicine	23
Pain and Therapy	21
Annals of Cardiac Anaesthesia	17
European Journal of Pain	16
Pain Research and Management	16
Anesthesiology Research and Practice	13
Journal of Palliative Medicine	13
Brazilian Journal of Anesthesiology (English Edition)	12
International Journal of Obstetric Anesthesia	12
JAMS Journal of Acupuncture and Meridian Studies	12
Scandinavian Journal of Pain	12

Seminars in Arthritis and Rheumatism	12
Neuromodulation	11
Palliative Medicine	11
Saudi Journal of Anaesthesia	11
Journal of Neurosurgical Anesthesiology	10
Middle East Journal of Anesthesiology	10
Trends in Anaesthesia and Critical Care	10
Acta Anaesthesiologica Belgica	9
Agri	8
Perioperative Care and Operating Room Management	8
Journal of Cellular and Molecular Anesthesia	7
Korean Journal of Pain	7
Pain Medicine	7
Anaesthesiology Intensive Therapy	6
Critical Care and Resuscitation	6

Journal of Headache and Pain	6
Journal of Perioperative Practice	6
Medical Gas Research	6
British Journal of Pain	5
Frontiers in Pain Research	5
Journal of Oral and Facial Pain and Headache	5
Ulusal Travma ve Acil Cerrahi Dergisi	5
AANA Journal	4
Anestezi Dergisi	4
Anesthesia Progress	4
Bali Journal of Anesthesiology	4
Pain Reports	4
Revista Espanola de Anestesiologia y Reanimacion	4
Canadian Journal of Anaesthesia	3
Canadian Journal of Pain	3

Journal of Research in Clinical Medicine	3
Local and Regional Anesthesia	3
Open Anesthesia Journal	3
Pain Management	3
Ambulatory Surgery	2
Annals of Critical Care	2
Brazilian journal of anesthesiology (Elsevier)	2
European journal of pain (London, England)	2
Journal of Pain and Palliative Care Pharmacotherapy	2
Southern African Journal of Anaesthesia and Analgesia	2
Sri Lankan Journal of Anaesthesiology	2
Anesthesia and Pain Medicine	1
Archives of Anesthesiology and Critical Care	1
Asian journal of anesthesiology	1

Asian Journal of Anesthesiology	1
BJA Open	1
DOLOR	1
JA Clinical Reports	1
Journal of Anesthesia, Analgesia and Critical Care	1
Journal of Neuroanaesthesiology and Critical Care	1
Journal of Opioid Management	1
Patient Safety in Surgery	1
Regional Anesthesia and Acute Pain Management	1
Revista de la Sociedad Espanola del Dolor	1
Revista Mexicana de Anestesiologia	1
Russian Journal of Anesthesiology and Reanimatology /Anesteziologiya i Reanimatologiya	1
Seminars in Cardiothoracic and Vascular Anesthesia	1

The journal of headache and pain	1
Therapeutic Hypothermia and Temperature Management	1

Sensitivity analysis - **Logistic Regression Analysis Identifying Risk Factors for Baseline Characteristics Assessment Removing Journals with Consistent Policies.** *Baseline characteristics statistical testing as the dependent variable.*

**Variable**	**OR(95%CI)**	**p value**	**Multicollinearity (VIF)**
Year	1.07(1.06 - 1.08)	<0.001	1.04
Journal Quartile			1.02
• First Quartile	reference		
• Second Quartile	1.44 (1.13 - 1.82)	0.003	
• Other Quartiles	2.18 (1.78 - 2.68)	<0.001	
Groups	0.76 (0.66 - 0.87)	<0.001	1.02
Authors	0.95 (0.93 - 0.98)	<0.001	1.07
Study population	1.00 (1.00 - 1.00)	0.307	1.01
Number of variables	0.98 (0.97 - 1.00)	0.017	1.08

CI: Confidence Interval, OR: Odds ratio, VIF: variance inflation factor
'Year' indicates the year of publication; 'groups' refers to the number of groups analyzed in the trial; 'authors' denotes the number of authors of the article; 'study population' represents the sample size included in the trial; and 'number of variables' corresponds to the number of baseline characteristics reported in the article
